# Detached, wetted strawberries take up substantial water in the calyx region

**DOI:** 10.1038/s41598-023-31020-0

**Published:** 2023-03-08

**Authors:** Grecia Hurtado, Moritz Knoche

**Affiliations:** Institute of Horticultural Production Systems, Leibniz University Hanover, Herrenhäuser Straße 2, 30419 Hannover, Germany

**Keywords:** Physiology, Plant sciences

## Abstract

In strawberry, surface disorders like ‘water soaking’, ‘cracking’ and ‘shrivel’ impair fruit quality of this high value crop. Water movement through the fruit surface is implicated a role in these disorders. The objective was to identify the pathways of water uptake and water loss (transpiration) and to identify factors affecting these flows. Water movement was quantified gravimetrically in detached fruit. Cumulative transpiration and uptake increased linearly with time. During ripening, fruit osmotic potential and water potential became slightly more negative. Rates of transpiration and water uptake and their corresponding permeances were constant during early ripening but increased as the fruit turned red. The permeance for osmotic water uptake was more than 10-times that for transpiration. Sealing selected regions of the fruit surface with silicone rubber allowed identification of the petal and staminal abscission zones in the calyx region and cuticular microcracks of the calyx region and receptacle as high flux pathways particularly for water uptake (osmotic). These results were confirmed by acridine orange infiltration and fluorescence microscopy. Increasing the relative humidity (RH) decreased the rate of transpiration, while increasing temperature increased both transpiration and water uptake. There was no effect of storing fruit (2 °C, ~ 80% RH) for up to 10 days. Our results identify petal and staminal abscission zones and cuticular microcracks as high flux pathways for water uptake.

## Introduction

Strawberry is a highly perishable, high-value fruit crop that is grown worldwide. The fruit is subject to rapid deterioration preharvest in the field and postharvest during transit, storage, and sale. Surface disorders such as water soaking, cracking, loss of shine and shrivel impair the fruit quality causing significant economical losses^1–4^. Water movement through the fruit surface, i.e. uptake into the fruit and water loss by transpiration from the fruit, is implicated a role in these disorders. Water soaking results from localized water uptake through microscopic cracks (‘microcracks’) on the fruit surface causing a bursting of cells. Microcracks are minute cracks in the cuticle invisible to the naked eye that do not extend into the underlying epidermis^5,6^. As a consequence, the fruit skin develops irregular, pale and deliquescent patches^7^. The macroscopic cracking of strawberries has not been studied in detail, but field observations suggest that water uptake after rainfalls may be involved^8^. Loss of shine, reduced calyx apperance^3,9^ and shrivel^10^ are typical symptoms of reduced turgidity as a result of water loss by transpiration.

Despite of its importance, little is known about the movement of water through the surface of strawberries. The mechanisms of transpirational water loss and osmotic water uptake have only recently been identified^6^. However, the predominant pathways of transpirational water loss and osmotic water uptake are largely unknown, nor have the external factors been identified that affect the rates of these water exchanges through the fruit surface.

The objective of our study was to identify the dominant pathways of water movement through the strawberry fruit surface, and also the external factors affecting the rates of those water movements.

## Results

**Figure 1:**
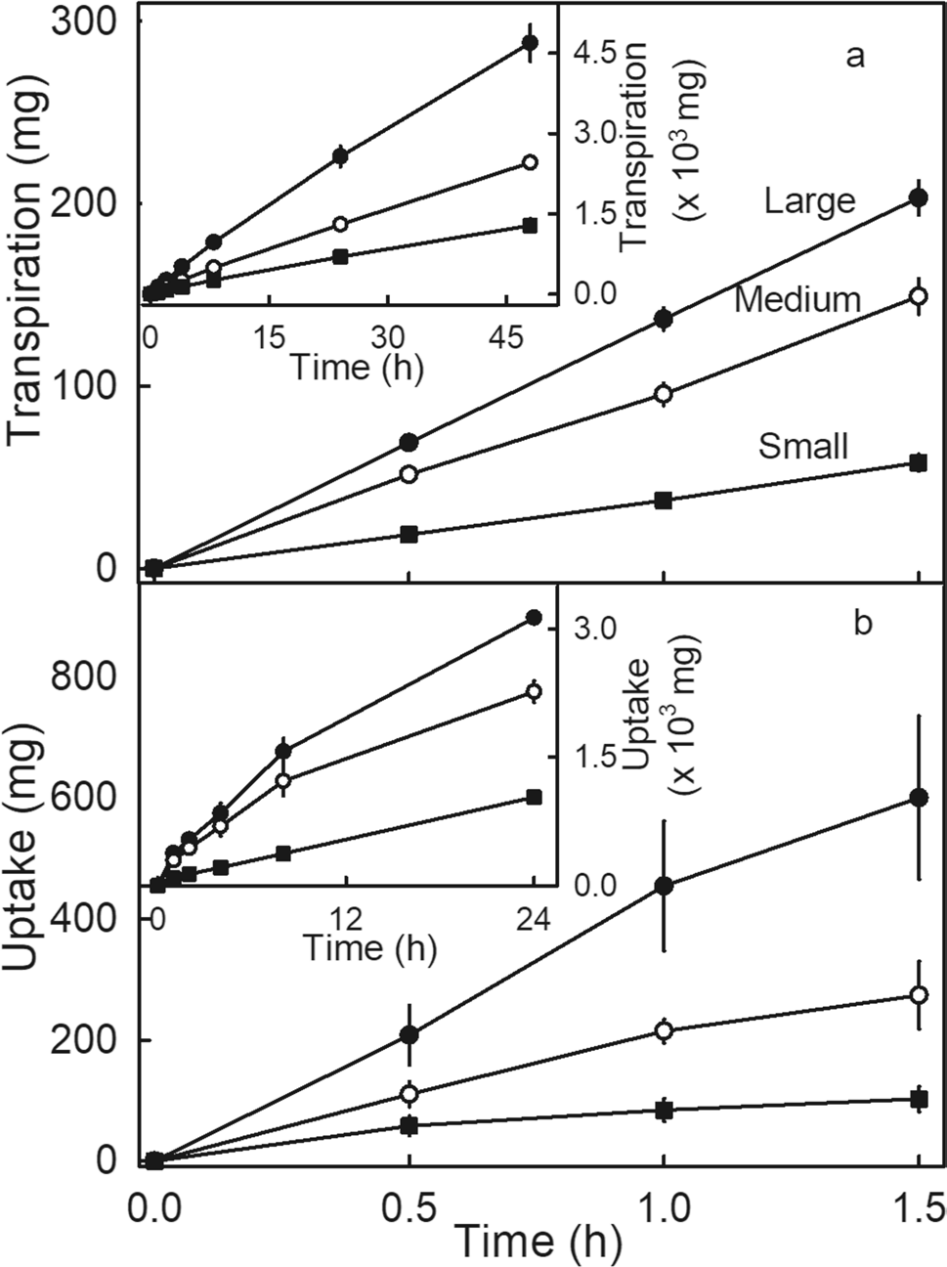
Short-term (main graph) and long-term (insets) time course of (**a**) transpiration and (**b**) water uptake of large, medium, and small strawberry fruit (cv. Clery).

Transpiration increased linearly with time indicating constant rates in both the short and the long-term for fruits of all size classes (Fig. 1a). For osmotic water uptake, uptake increased linearly with time only until about 8 h when uptake rates decreased slightly for the medium- and large-size fruit (Fig. 1b-inset). Uptake rates were generally more variable than transpiration rates. Both transpiration and uptake rates were positively related to fruit size (Fig. 1a,b).

**Figure 2:**
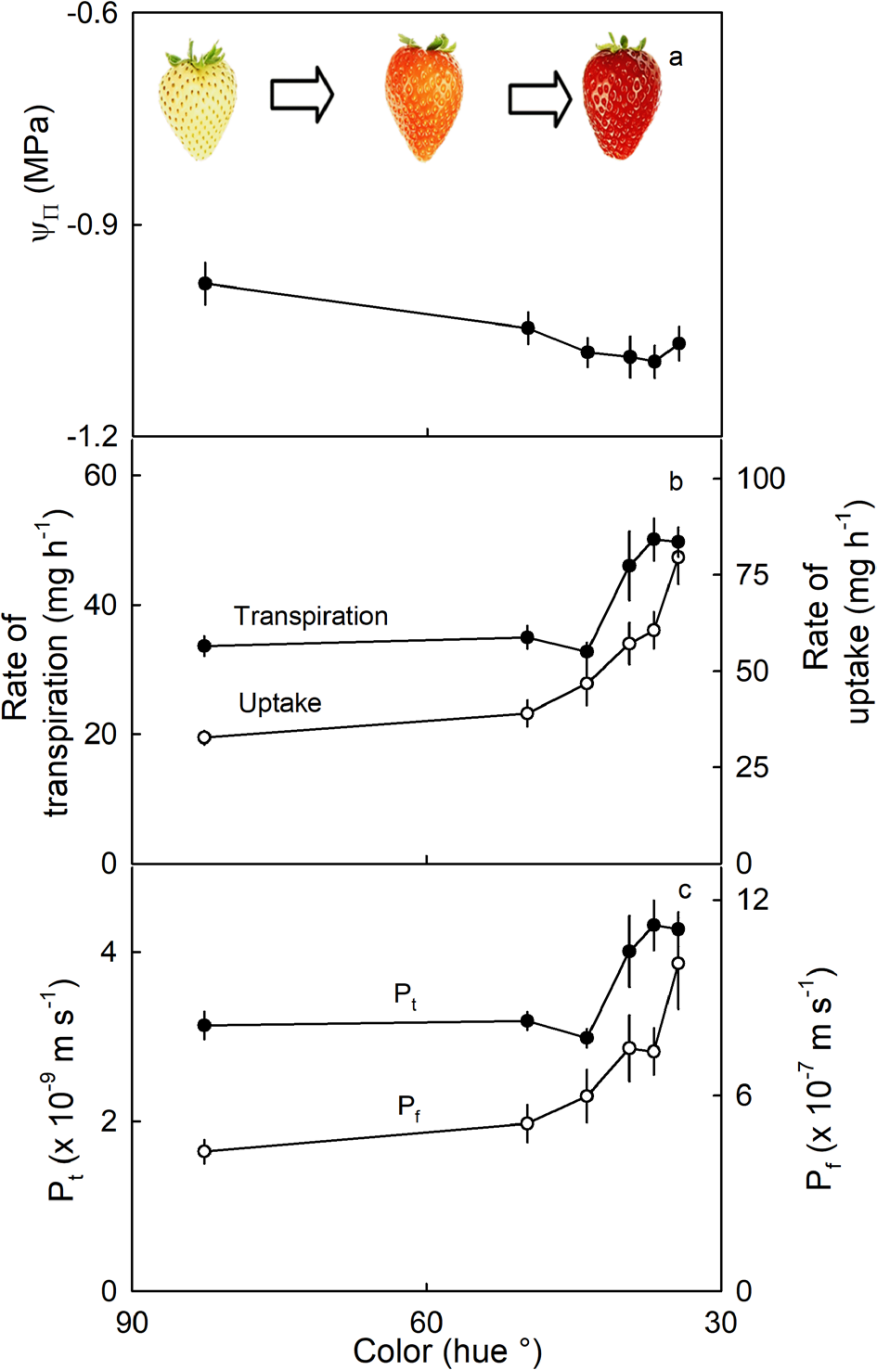
Changes during ripening of strawberry fruit indexed by (**a**) osmotic potential (${\Psi }_{\Pi }$), (**b**) the rates of transpiration and uptake, and (**c**) the permeances for transpiration (P_t_) and water uptake (P_f_) of ‘Florentina’ strawberry. The time of ripening was indexed by the change in color.

Transpiration and water uptake changed with ripening (Fig. 2). As ripening progressed, as indexed by a decrease in hue angle from white (82.7° hue) to dark red (34.3° hue), the osmotic potential of the expressed fruit juice and the water potential of the whole fruit became slightly more negative (Fig. 2a). The rates of transpiration and water uptake were constant up to a hue angle of about 45° then increased as the fruit turned red (Fig. 2b). Estimating the fruit surface area from fruit shape and assuming conical shape allowed calculation of the permeance of the fruit surface. The permeances for both transpiration and water uptake increased as the fruit turned red (Fig. 2c). The permeance values for transpiration were consistently lower than those for osmotic water uptake, by more than an order of magnitude, irrespective of the stage of ripening.

Preferential pathways for water movement are pathways, where rapid movement occurs through small cross-sectional areas. Consequently, these areas represent high flux pathways in regions of preferential water movement. To identify preferential pathways of transpiration and water uptake, several experiments were conducted comprising a complexity of treatments. The first experiment was a two-phase one, where the initial rates of water movement were established during phase I. Then, in phase II, parts of the fruit surface were selectively sealed or excised before incubation was continued and rates of water movement were re-evaluated. A second group of fruit remained untreated during phase II and served as the controls.

**Figure 3:**
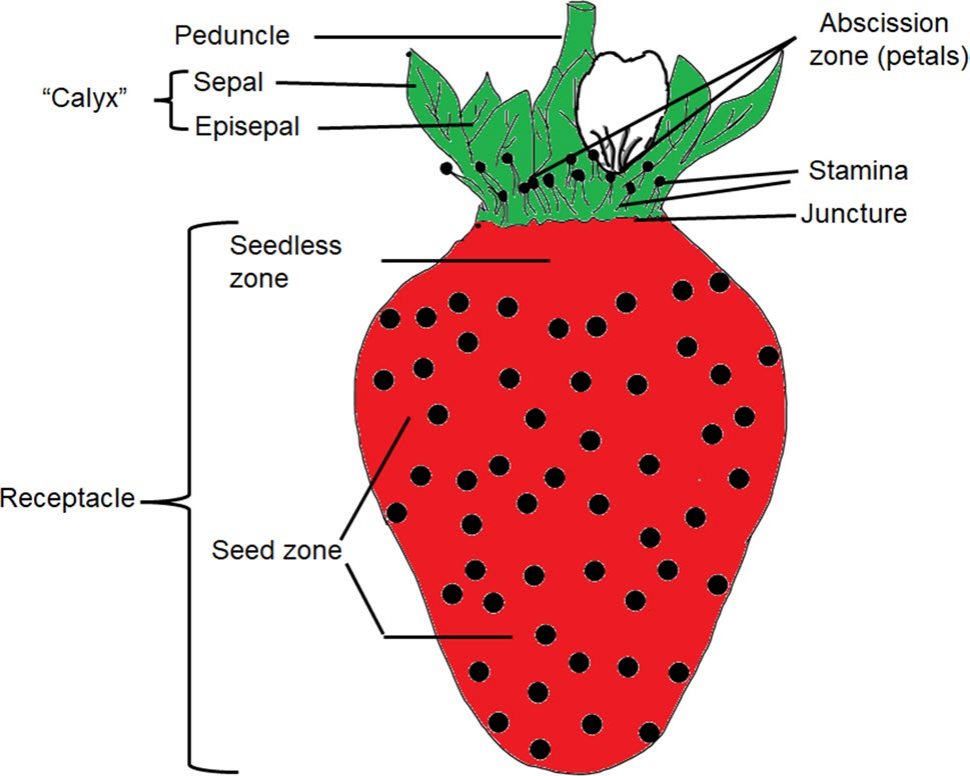
Sketch illustrating the nomenclature used to describe the different regions and organs of a strawberry fruit addressed by the sealing treatments. We refer to the portion of the receptacle that lacks seeds (strictly, the zone lacking the single-seeded achenes) as the ‘seedless’ zone.

**Figure 4:**
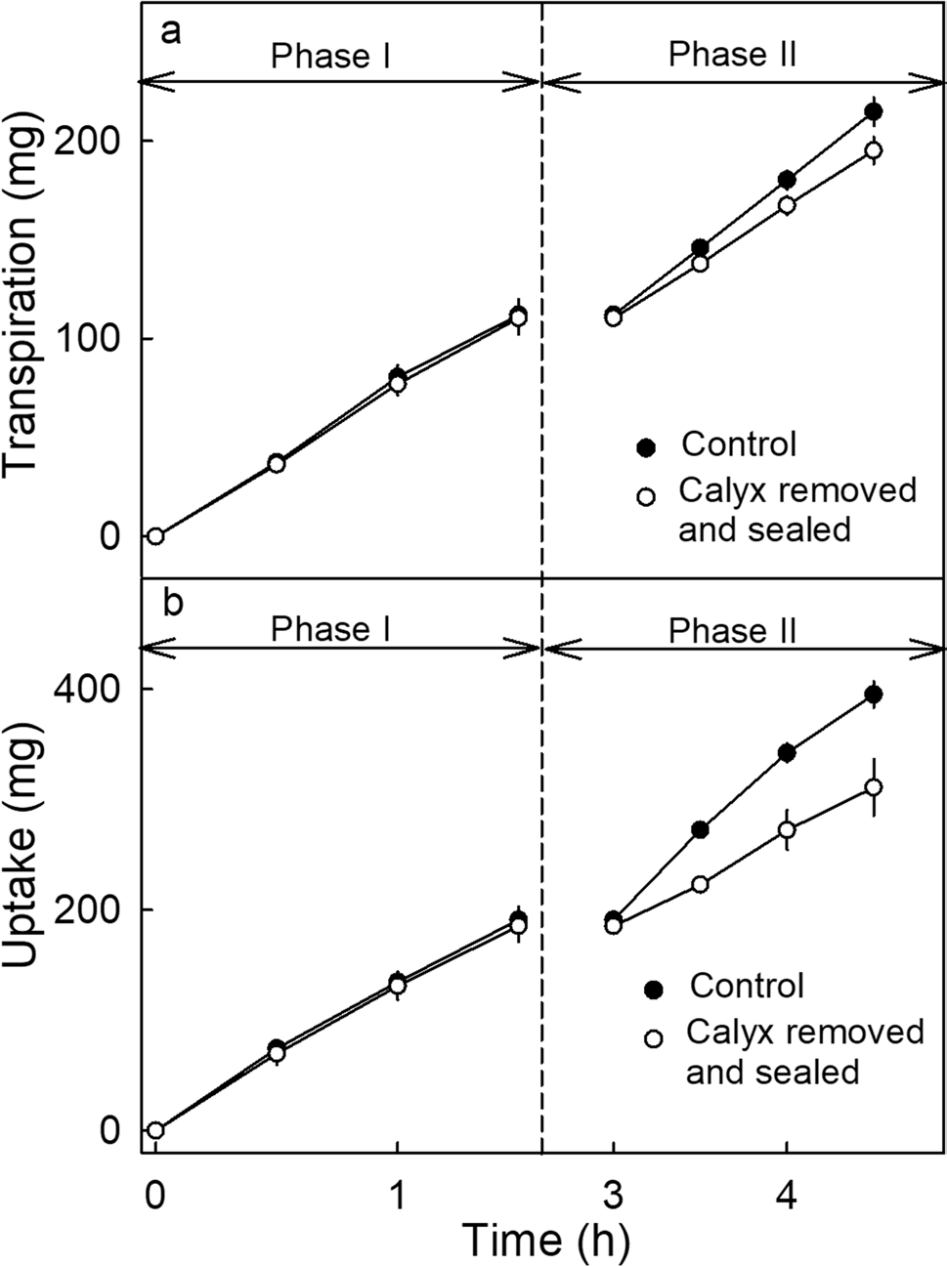
Effect of sealing selected regions of the surface of ripe strawberry cv. Sonsation on water movement in (**a**) transpiration or (**b**) water uptake. The experiment was conducted in two phases using a repeated measures design. During phase I (0 to 1.5 h), water movement was quantified in non-sealed fruit with calyx and 5 mm of the peduncle. For phase II (3–4.5 h), sepals and stamina were removed and the cut surface of the sepals, the abscission zones of the petals, the stamina, the junction calyx-receptacle, the peduncle surface including the peduncle end were all sealed (‘Calyx removed and sealed’). Fruit that remained without treatment during phase II served as control. For flow rates see Table. 1.

The first experiment focused on the calyx region. During phase I, both transpiration and water uptake increased linearly with time. During phase II, in one group of fruit, the entire calyx was excised including the abscission zones of petals and stamina, and the resulting wound was sealed using silicone rubber (Fig. 3). This treatment reduced the rate of transpiration and, even more, the rate of water uptake. Transpiration and water uptake in the untreated controls continued to increase (Fig. 4, Table 1). In the second comparison, the same treatments were applied for phase II but the cut surfaces (wounds) remained unsealed. Following excision without sealing, the rates of transpiration increased slightly, whereas the rates of water uptake more than doubled (Table 1). In the third comparison, we addressed the role of the cut peduncle end. Here, the cut end was sealed for phase II. It was found that sealing the cut peduncle end had no effect on either the rate of transpiration or the rate of water uptake (Table 1).

**Table 1:** Rates of water uptake and transpiration along different pathways of strawberry cv. Sonsation. The experiment was conducted in two phases using a repeated-measures design. During phase I (incubation for 0–1.5 h) uptake and transpiration were determined without any treatment (‘control’). During phase II (incubation in water for 3–4.5 h), the remains of floral parts were excised, or parts of the fruit surface sealed using silicone rubber. Phase II treatments comprised the excision of sepals and stamina and subsequent sealing of the cut surfaces, the calyx-receptacle junction and peduncle surface including the peduncle end (‘Control/sepals and stamina cut, and junction calyx-receptacle and peduncle end sealed’), the excision of sepals and stamen without subsequent sealing of the cut surfaces (‘Control/sepals and stamina cut, no seal’), and sealing of the cut peduncle end (‘Control/peduncle-end sealed’). Fruit without excision and without sealing served as controls (‘Control/control’).

Treatment	Rate of transpiration (mg h^−1^)			Rate of water uptake (mg h^−1^)		
Phase I/Phase II	Phase I	Phase II	Δ Rates	Phase I	Phase II	Δ Rates
Control/Control	75.8 ± 5.5	68.5 ± 4.8	−7.3 a^a^	126.7 ± 7.7	136.4 ± 7.9	9.8 a^a^
Control/sepals, abscission and stamina zone cut, and cut surface, junction calyx-receptacle, and peduncle end sealed	74.4 ± 5.5	56.7 ± 4.7	−17.7 b	123.5 ± 9.5	85.2 ± 17.8	−38.3 b
Control/Control	84.4 ± 6.5	82.2 ± 6.1	−2.2 a	129.7 ± 11.2	171.6 ± 17.1	41.9 a
Control/sepals, abscission and stamina zone cut no sealing	80.8 ± 6.5	92.7 ± 6.2	11.9 b	141.1 ± 12.3	376.3 ± 39.7	235.2 b
Control/Control	86.3 ± 5.9	85.3 ± 5.9	−1.0 a	149.4 ± 8.3	180.6 ± 7.5	31.2 a
Control/peduncle-end sealed	79.2 ± 3.8	76.7 ± 3.7	−2.4 a	160.6 ± 12.6	169.6 ± 21.6	9.0 a

^a^Mean separation within main effect by Dunnett’s test at *p* = 0.05. Each treatment was compared with its control.

In the second experiment on preferential water pathways, the portion of the fruit surface responsible for the increased water movement in the calyx region was identified by sealing a progressively increasing portion of the fruit surface—beginning at the proximal end of the fruit (Fig. 3). Selective sealing of the sepals and peduncle surface had almost no effect on the rate of transpiration but reduced the rates of osmotic water uptake by 13% (Table 2). When (in addition to the sepals) the abscission zones of the petals and stamina, and the junction between the calyx and receptacle were sealed, the rate of transpiration decreased slightly, however, the rate of water uptake decreased much more, by about 23% (Table 2). When the entire calyx was removed, including the sepals, the abscission zones, and the junction between calyx and receptacle, and the seedless zone was sealed, the rate of transpiration was reduced by about 25%, and the rate of water uptake by about 31% (Table 2).

**Table 2:** Rates of water movement for transpiration and osmotic uptake along different pathways through the strawberry fruit surface (cv. Sonsation). Fruits were progressively sealed, starting from the proximal end. Treatment **a** (‘Control’): Fruit without sealing served as control. The sealing treatments were: Treatment **b** (‘Sepals sealed’): sepals and the peduncle surface sealed, open remained the abscission zones of petals, stamina, the junction between calyx and receptacle, and the seedless zone of the receptacle. Treatment **c** (‘Sepals, abscission zones, stamina, and junction sealed’): sepals, abscission zones of petals and stamina, peduncle and junction calyx-receptacle cut end all sealed. Treatment **d** (‘Seedless zone sealed’): seedless zone of the receptacle, sepals, abscission zone of petals and stamina, calyx-receptacle junction, peduncle removed and sealed.

Treatment	Rate of transpiration		Rate of water uptake	
	(mg h^−1^)	(%)	(mg h^−1^)	(%)
**a**. Control	97.7 ± 6.0	100	258.7 ± 27.6	100
**b**. Sepals sealed	95.9 ± 3.6	98.2	225.5 ± 19.4	87.2
**c**. Sepals, abscission zone of petals, stamina, and junction sealed	91.2 ± 5.8	93.3	199.7 ± 18.0	77.2
**d**. Seedless zone sealed	72.9 ± 3.8	74.6	177.1 ± 15.2	68.5

From this experiment, the relative contributions of the various pathways of transpiration and water uptake could be calculated (Table 3). Most of the transpiration (93%) occurred through the surface of the receptacle, including that of the seedless zone. However, the receptacle and seedless zone together accounted for only 77% of water uptake. The remaining 23% of water uptake occurred in the region of the sepals, the petal and staminal abscission zones, and the calyx-receptacle junction (Table 3).

**Table 3:** Relative contribution of different pathways to whole fruit transpiration and water uptake into strawberry fruit cv. Sonsation. The pathways were: sepals, the peduncle surface, abscission zone of petals, stamina, the junction between calyx and receptacle, and the seedless zone of the receptacle. Data is calculated from data in Table 2.

Pathway	Rate of transpiration		Rate of uptake	
	(mg h^−1^)	(%)	(mg h^−1^)	(%)
Sepals and peduncle surface	1.8	1.9	33.2	12.8
Abscission zone, stamina, junction calyx-receptacle,	4.7	4.8	25.8	10.0
Seedless zone	18.2	18.7	22.6	8.7
Receptacle (with achenes)	72.9	74.7	177.1	68.5
Whole fruit	97.7	100	258.7	100

The distinct role of the water uptake pathways in the various structures located at the proximal end of the fruit (the petal and staminal abscission zones, and the calyx-receptacle junction) was not unique to ‘Sonsation’ but was also observed in the other cultivars examined (Table 4). Only in ‘Elsanta’ was the magnitude of the contribution to total water uptake made by these pathways almost negligible. It is worth noting here that the rates of osmotic water uptake differed significantly among the cultivars examined by a factor of 4. Rates were highest in ‘Clery’ and lowest in ‘Joly’ (Table 4).

**Table 4:** Rates of osmotic water uptake into detached strawberries of selected cultivars. Water uptake was quantified on a whole fruit basis and after sealing abscission zones of petals and stamina, and junction calyx-receptacle (‘abscission zone and junction sealed’) using silicone rubber. The contribution of the abscission zone and the junction was calculated by subtracting the uptake in the sealing treatment from that of the non-sealed control. Non-sealed fruit served as control.

Cultivar	Rates of uptake			
	Whole fruit	Whole fruit with abscission zone and junction sealed	Abscission zone and junction	
	(mg h^−1^)	(mg h^−1^)	(mg h^−1^)	(%)
Sonsation	208.2 ± 17.0	134.7 ± 9.0	73.5	35.3
Clery	349.9 ± 51.7	254.6 ± 25.3	95.4	27.3
Florentina	127.8 ± 5.1	81.7 ± 3.9	46.1	36.1
Dream	86.6 ± 13.3	70.5 ± 9.2	16.1	18.6
Joly	115.1 ± 17.4	83.7 ± 6.7	31.4	27.3
Elsanta	221.9 ± 28.8	212.1 ± 34.3	9.8	4.4
Grand Mean	181.0 ± 14.3a	141.2 ± 9.8 b	45.4	24.8

^a^Mean separation within main effects by Tukey’s test, P = 0.05. No interaction between main effects was found by ANOVA analysis.

**Figure 5:**
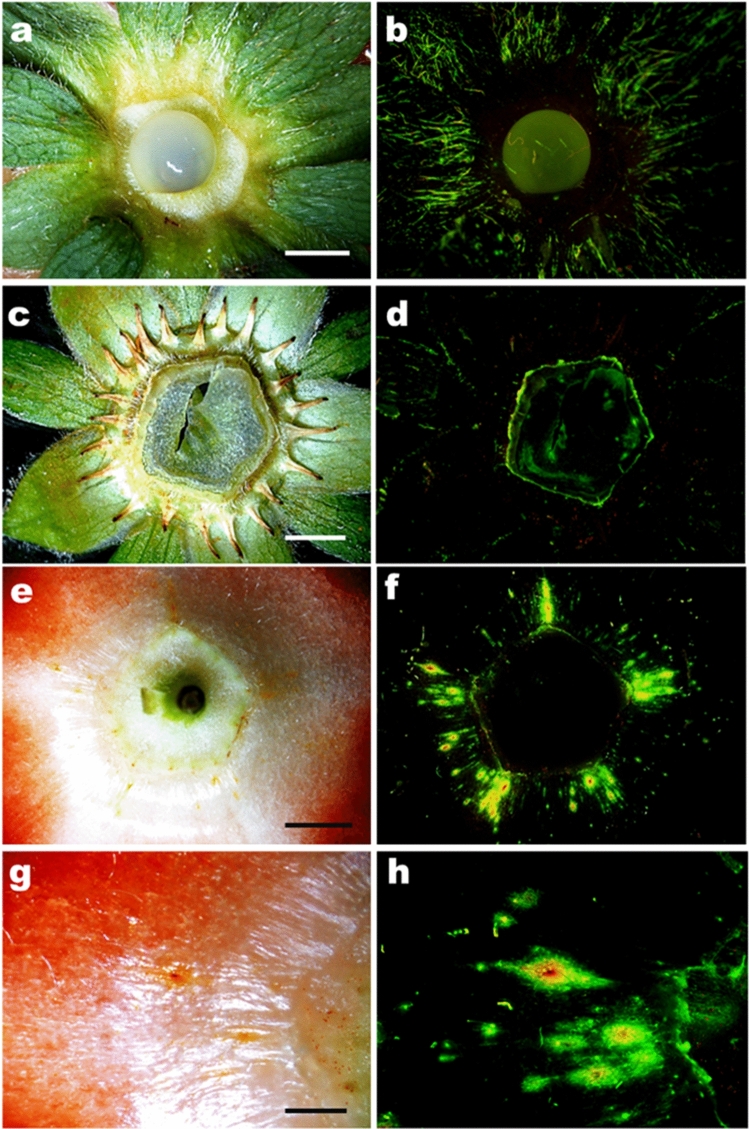
Micrographs of the calyx region of ripe strawberries cv. Sonsation after incubation in the fluorescent tracer acridine orange. Images were taken in incident bright light (**a, c, e, g**) and incident fluorescent light (**b, d, f, h**). **a, b** abaxial surface of sepals and episepals with numerous trichomes stained by acridine orange. The opaque dot in the center is a drop of silicone rubber that was applied for sealing the peduncle end; **c, d** adaxial surface of sepals and episepals; **e, f** top view of the seedless zone of the receptacle below the calyx showing fluorescing microcracks in the vertices of the pentagon-shaped base; **g, h** same as **e, f** but microcracks at higher magnification. Scale bar in a,c,e = 3 mm, and g = 1 mm.

**Figure 6:**
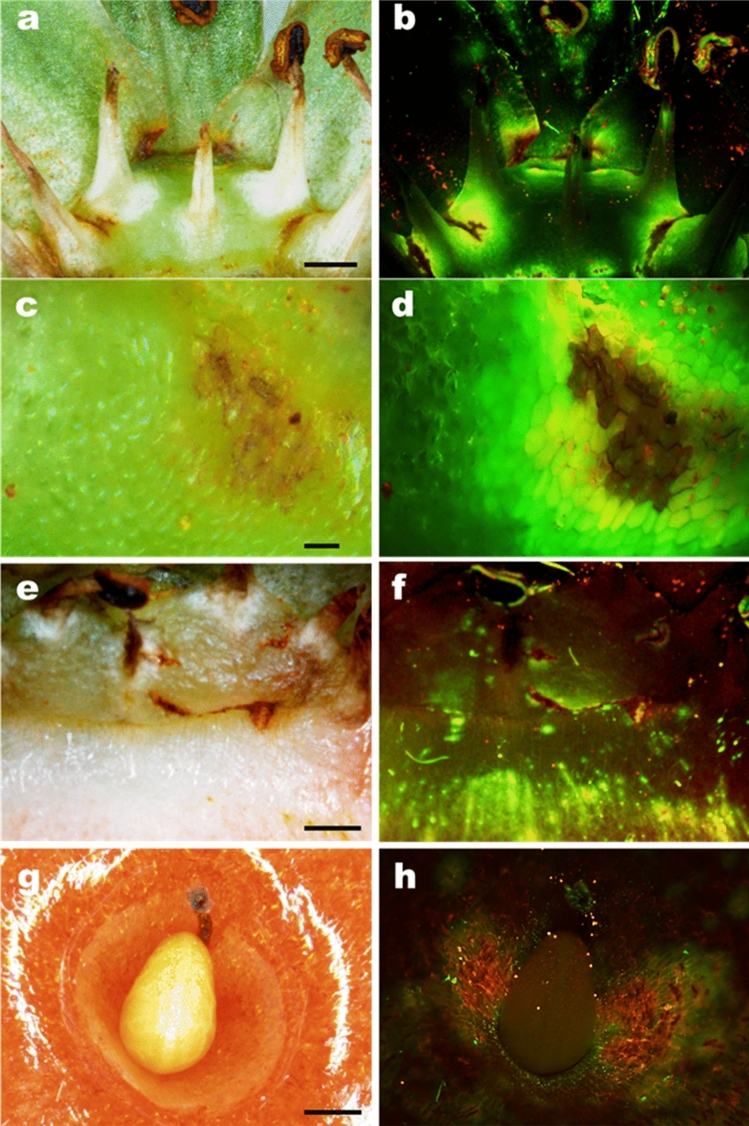
Micrographs of microcracks in the cuticle on the surface of ripe strawberries cv. Clery after incubation in the fluorescent tracer acridine orange. Images were taken in incident bright light (**a, c, e, g**) and incident fluorescent light (**b, d, f, h**). **a, b** microcracks at the base of stamina and the abscission zone of petals; **c, d** detailed view of microcrack at the base of a stamen; **e, f** microcracks at the calyx-receptacle junction and in the seedless zone of the receptacle; **g, h** microcracks in the depression of an achene on the surface of strawberry fruit. Scale in a,e = 1 mm, c = 0.1 mm and g = 0.5 mm.

Acridine orange dye and fluorescent microscopy were used to identify in more detail the sites of water uptake. This revealed staining of the trichomes on the sepals and episepals (Fig. 5a–d). Trichome staining was more pronounced on the abaxial as compared to on the adaxial surfaces (Fig. 5a, b). The base of the calyx on the receptacle was pentangular in shape. Dye infiltrated microcracks were clearly visible; these extended radially from the vertices of the pentagon up into the seedless zone of the receptacle (Fig. 5e–h). Infiltration was also observed in the petal abscission zones and in the bases of some stamina (Fig. 6a, b). Dye infiltration resulted from microcracks in the region of the staminal bases (Fig. 6c, d) and the calyx-receptacle junction (Fig. 6e, f). Numerous microcracks also occurred in the depressions in which lay the seeds on the receptacle (Fig. 6g, h).

**Figure 7:**
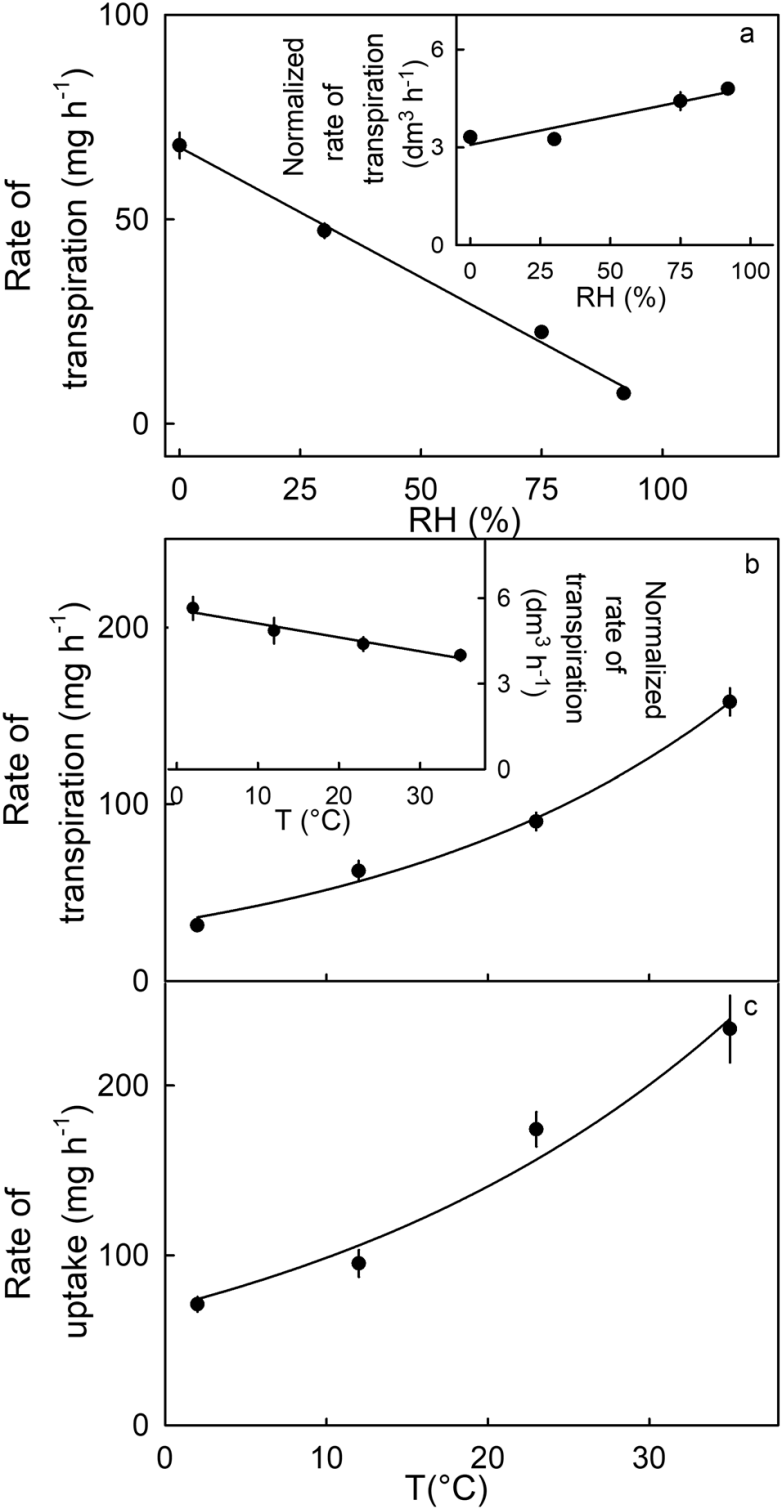
(**a**) Effect of relative humidity (RH) on the rate of transpiration (main graph) and on the normalized rate of transpiration (inset). (**b**) Effect of temperature (T) on the rate of transpiration (main graph) and on the normalized rate of transpiration (inset) and (**c**) on the rate of water uptake of ‘Laetitia’ strawberry. Normalized transpiration rates were calculated by dividing the rate of transpiration by the difference in water vapor concentration between the inside of the fruit^20^ – assumed to be a saturated atmosphere – and the outside atmosphere above dry silica gel – assumed to be 0 g m^−3^.

Increasing the relative humidity (RH) decreased the rate of transpiration (r^2^ = 0.98***). Fruit incubated at high humidity had the lowest rates of transpiration (Fig. 7a-main graph). When the recorded rates of transpiration were normalized for the driving force at each of the different humidities, the rates became nearly independent of humidity. This indicates that skin permeance was largely unaffected by the RH (Fig. 7a-inset).

Increasing the temperature increased the rates of transpiration and water uptake (Fig. 7b, c). The increase was exponential. Varying the temperature also varied the driving force for transpiration and water uptake. After normalizing for the different driving forces, the rates of transpiration decreased slightly but the relationship between normalized transpiration rate and temperature was not significant (Fig. 7b-inset). This indicates that skin permeance to water was also largely unaffected by temperature.

**Figure 8:**
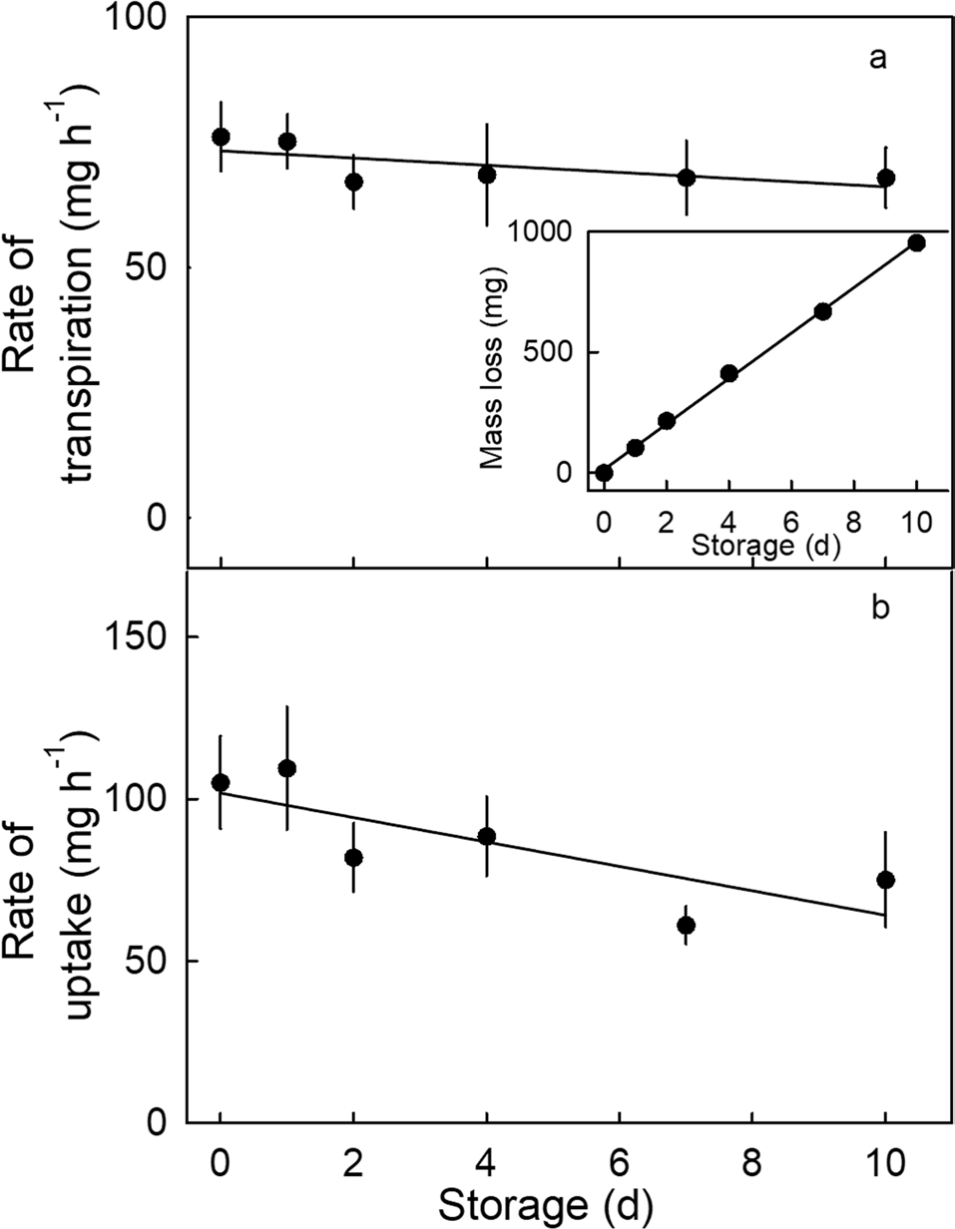
Effect of cold storage duration on (**a**) the rate of transpiration (main graph), mass loss (inset), and (**b**) the rate of water uptake of ‘Clery’ strawberry following storage.

**Figure 9:**
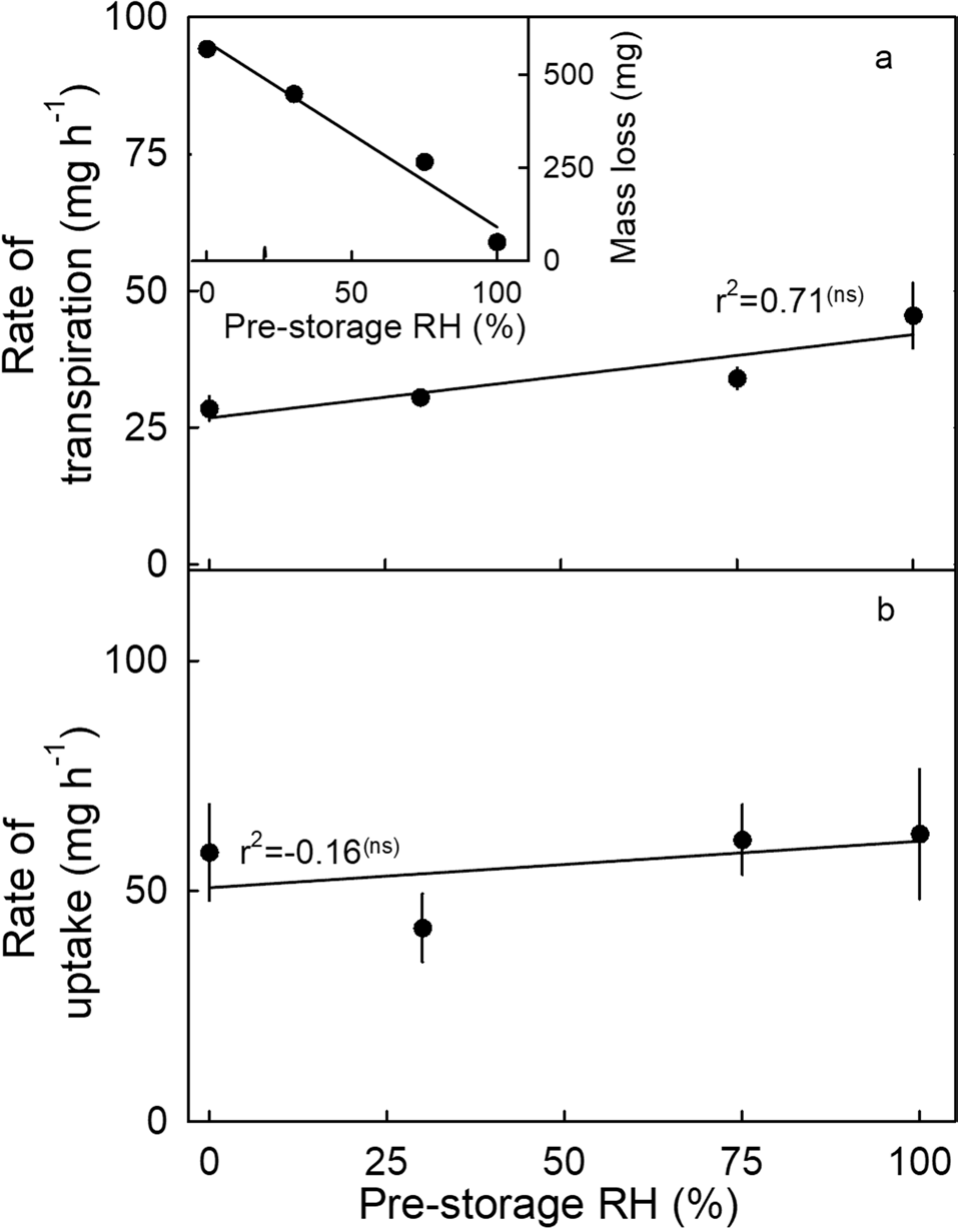
Effect of relative humidity (RH) during a 24 h pre-storage period on (**a**) the rate of transpiration (main graph) and cumulative mass loss (inset) and on (**b**) the rate of uptake of ‘Clery’ strawberry.

There were no significant effects of holding fruit in storage at 2 °C and ~ 80% RH for up to 10 days on the rates of transpiration or osmotic water uptake (Fig. 8). During storage, the total mass loss increased linearly with time, reached about 5% of the initial fruit fresh mass by 10 d (Fig. 8-inset). Cumulative mass loss had no effect on the rate of mass loss. Similarly, holding fruit for 24 h under different conditions of RH at 22 °C had no significant effect on the rates of water loss or uptake (Fig. 9).

## Discussion

Our results indicate two general findings. (1) That the water fluxes into and out of a strawberry fruit occur along a number of distinct parallel pathways. Of these pathways and flows, preferential water movements occur in the calyx region, particularly for osmotic water uptake. And (2) both transpirational loss and osmotic water uptake exhibit behaviors typical of purely physical processes—there being no evidence of strong temperature sensitivity nor of responsive changes in water permeance.

### Preferential parallel pathways for water uptake

Our study identified several of parallel pathways through which in particular osmotic water uptake occurred. The preferential high flux pathways were: (1) the junction region between calyx and receptacle, (2) the abscission zones of the petals and stamina, and (3) distinct microcracks in the receptacle. Compared to the entire fruit surface, these pathways have a small cross sectional area for water uptake and therefore represent high flux pathways. The above conclusions are based on the following observations.

First, selective sealing of these areas with silicone rubber markedly reduced osmotic water uptake. Conversely, when the calyx region was excised without sealing, osmotic water uptake increased markedly indicating that a strong driving force for uptake was present and that the vasculature in this region was still functional.

Second, fruit incubated in a solution of the fluorescent tracer acridine orange revealed various pathways for rapid water uptake that 'bypassed’ the cuticle on the fruit surface. Acridine orange does not penetrate the cuticle, so tissue penetration must be via openings in the cuticle. The pathways for tissue penetration include microcracks: in the seed depressions on the receptacle; in the seedless zone; at the calyx-receptacle junction; and at the bases of the staminal and petal abscission zones. It is speculated that enhanced penetration in the abscission zones is due to the associated vascular bundles remaining open following the abscission. The role in water uptake of the trichomes on the abaxial surface of the calyx is unclear. The trichomes were stained and occasionally also some epidermal cells at the trichome bases. This indicates that dye penetration occurred. Thus, trichomes are also sites of preferential entry of water. The proportional contribution of water uptake at trichomes to total water uptake by the fruit is not known. Water uptake along these pathways is relatively rapid because the cuticle is bypassed as a penetration barrier.

The causes of microcracking in strawberries are unknown. Potential explanations include: (1) The development of cuticular growth strains during organ development^11,12^. Such strains occur particularly when cuticular deposition rates fail to keep pace with surface area expansion rates during organ development^13^. And that: (2) The microcracking of an already-strained cuticle is exacerbated by surface wetness as has been shown in a number of fruit crops species including sweet cherry, apple, mango, and grape^14–17^. In spite of the botanical distinction (a strawberry is not a true fruit) research shows that in many ways its growth and development are more closely similar to those of a true fruit than they are to a floral accessory organ, a receptacle. We note that the region of the calyx-receptacle junction is likely to suffer from extended periods of surface wetness making cuticular microcracking all the more likely.

### Transpiration and water uptake are physical processes

Transpiration and water uptake are physical processes that do not require metabolic energy and so are relatively temperature insensitive. We found that water movement followed a general transport equation where the rate of movement is a function of the cross-sectional area through which the movement occurs, the driving force that causes the movement, and the ease of movement—a hydraulic conductance term—i.e., the permeance of the fruit skin or a porous pathway within it. This conclusion is inferred from the following findings.

First, rates of transpiration and water uptake were higher for larger fruit compared to for intermediate or small fruit. This is to be expected since the cross-sectional area through which movement occurs is greater in larger fruit. Second, the rate of transpiration was strongly dependent on RH and temperature, while those of water uptake were dependent on temperature only. When accounting for differences in driving force between different temperatures and different RHs, the normalized flow rates were proportional to the skin permeances and nearly independent of temperature and RH. Third, the lack of any effect of pre-storage duration is consistent with the concept of a physical transport process.

Earlier studies have established that the cuticle forms the rate-limiting barrier in water movement^6^. Effects on properties that affect cuticle permeance during a 10-d cold storage period are highly unlikely. For example, it might be argued that the mass loss that occurred during storage should have had an effect on the normalized flow rates. However, the mass loss during storage did not exceed 5% of the fruit’s initial fresh mass. In a first approximation, this would yield a decrease in osmotic potential of about 5%. The anticipated increase in osmotic water uptake rate (+ 5%) would be too small to have been detected in our experiments.

The conclusions drawn here are also consistent with earlier reports. Transpiration occurs primarily by gaseous diffusion. In osmotic water uptake, however, viscous flow along ‘porous pathways’ is expected to occur in parallel to diffusion through the cuticle^18^. In strawberries, these parallel pathways include microcracks, abscission zones, a leaky junction between receptacle and calyx, and (possibly) ruptured stomata and broken trichomes. Viscous flow through such openings is rapid because it bypasses the cuticle as the primary barrier. The occurrence of microcracks on the fruit surface is highly variable and highly localized within a fruit. Rates are also highly variable between fruit on the same parent plant or between fruits on neighboring plants in the same planting. This may explain the variability in rates of osmotic water uptake by fruit that typically exceeds that reported for transpiration. Viscous flow through these openings also accounts for the higher permeance for osmotic water uptake, as compared to that for transpiration in both this and an earlier study^6^.

## Conclusion

Our study provides evidence for multiple and high flux pathways for osmotic water uptake into strawberry fruit. Water uptake along these pathways occurs by viscous flow. Uptake by viscous flow is rapid compared to diffusion through an intact cuticle. Potential consequences of this rapid uptake are the development of macroscopically visible cracks resulting in cracked fruit and, most likely, the initiation and subsequent spreading of water soaking on the fruit surface. In addition, macroscopic and microscopic cracks account for the high susceptibility of strawberries to fruit rots. Since microcracks are exacerbated by surface water, the avoidance of surface wetness is key to reducing fruit rots and increasing fruit quality. These benefits can be expected to be enhanced if strawberry production is moved from field to protected cultivation.

## Material and methods

### Plant material

Strawberry (*Fragaria* × *ananassa* Duch.) fruits were harvested from commercial plantings at Gleidingen (lat. 52°16′N, long. 9°50′E), Ohndorf (lat. 52°21′N, long. 9°21′E), a growth chamber on the Herrenhausen Campus (lat. 52°23′N, long. 9°42′E), and the Horticultural Research Station of the Leibniz University in Ruthe, Germany (lat. 52°14′N, long. 9°49′E). Temperature and relative humidity (RH) of the growth chamber were set at 20/16 °C and 60/80% RH during a 16/8 h day/night photoperiod. Fruits of the following cultivars were used: Clery, Sonsation, Florentina, Laetitia, Dream, Joly, and Elsanta. The use of different cultivars was necessary since strawberries at the optimum ripening stage are available only for a short period of time. The change of cultivars ensured that the effects observed are valid for strawberry in general and not limited to a specific cultivar.

Fruit was harvested randomly at commercial ripeness (> 80% of the fruit surface red)^19^. Uniform fruit was selected based on size, color, and freedom from visual defects. Unless specified otherwise, fruit was processed fresh on the day of collection or after a maximum of 2 d of storage at 2 °C and 80% RH.

### Transpiration and uptake

In most of the experiments, the calyx (sepals, episepals), abscission zones of the petals, and stamina (Fig. 3) were carefully removed from the fruit, and the site of attachment of the calyx to the peduncle was sealed using a fast-curing silicone rubber (Silicone rubber, SE 9186 Clear; Dow Corning Corp., Midland, USA). All experiments were carried out in a temperature-controlled laboratory at 22 °C. The number of individual fruit replicates was 15 unless otherwise specified.

For transpiration experiments, fruits were held in a polyethylene (PE) box above silica gel (RH ~ 0%;^20^) for 1.5 h, unless specified otherwise. Fruit was removed from the box and weighed individually at 30 min intervals. The rate of transpiration (F_t_; mg h^−1^) was calculated as the slope of a linear regression line of the relationship between the change in fruit mass and time.

For osmotic water uptake experiments, fruits were incubated individually in beakers (150 mL) with deionized water. A soft foam plug was placed on the beakers to sink the fruit, so as to wet the whole fruit surface. Fruits were removed from water and carefully blotted using soft tissue paper and then weighed at 30-min intervals for 1.5 h. The osmotic water uptake rate represented the change in fruit mass measured on an individual fruit basis. The rate of osmotic uptake (F_f_; mg h^−1^) was calculated as the slope of a linear regression line fitted through the relationship of change in fruit mass and time.

#### Time course of water movement

Short- and long-term time courses of osmotic water uptake and transpiration water loss were established for large (> 32 g), medium (15 to 32 g), and small (< 15 g) fruit of cv. Clery (Fig. 1). For the short-term measurements, fruit were weighed at intervals of 30 min for up to 1.5 h and for the long-term time course at 0, 1, 2, 4, 8, 24 h, and for transpiration up to 48 h.

#### Effect of ripening on water movement

The effects of ripeness on osmotic water uptake and transpirational water loss were identified using six stages of ripeness (Fig. 2). The ripeness stages were: white, 1/2 light red, 3/4 light red, 1/2 red, 3/4 red, dark red^21^. Fruits of ‘Florentina’ were selected based on color (CM-2600 d, orifice 3 mm diameter; Konica Minolta, Tokyo, Japan). The fruit selected ranged from white to dark red. Color was expressed as the hue angle. Rates of water uptake and transpiration were determined as indicated above. Additionally, juice was extracted from the fruit using a garlic press and its osmotic potential quantified by vapor pressure osmometry (VAPRO 5600; Wescor, Utah, USA). The skin permeances for transpiration (P_t_; m s^−1^) and osmotic water uptake (P_f_, m s^−1^) were determined from rates of water movement^22^. Briefly, P_t_ was calculated from Eq. (1). The rate of transpiration (F_t_; kg s^−1^) was divided by the product of the fruit surface area (A; m^2^), the density of water (ρ_w_; kg m^−3^), and the gradient in water activity (Δɑ_w_; dimensionless) across the fruit skin^23^. Since the humidity above dry silica is practically zero, Δɑ_w_ equals the water activity of the strawberry juice, which is approximately one.

The value of P_f_ (m s^−1^) was determined using the filtration permeability relation in Eq. (2); where F_f_ (kg s^−1^) represents the rate of osmotic uptake, A_fruit_ the fruit surface area (m^2^), R (m^3^ MPa mol^−1^ K^−1^) the universal gas constant, T (K) the absolute temperature, V_w_ (m^3^ mol^−1^) the molar volume of water and ρ_w_ (kg m^−3^) the density of water and ΔΨ (MPa) the difference in water potential between the water potential of the fruit (Ψ_fruit_) and that of the incubation solution (Ψ)^24^. For fruit incubated in water (Ψ = 0) the driving force for osmotic uptake is essentially equal to the water potential of the fruit (Ψ_fruit_). The fruit water potential equals the sum of the fruit’s turgor and the osmotic potential of the expressed juice (Ψ_Π_). Because the fruit turgor is negligibly low in strawberry^6^, the value of Ψ_Π_ essentially equals the Ψ_fruit_.

1 $${P}_{t}=\frac{{F}_{t}}{{A}_{fruit} \cdot {\rho }_{w}\cdot \Delta {a}_{w}}$$

2 $${P}_{f}=\frac{{F}_{f}}{{A}_{fruit} \cdot \Delta\Psi }\cdot \frac{RT}{\rho \cdot \overline{{V }_{w}}}$$

Fruit surface area was calculated from a solid geometrical model comprising a truncated cone capped by two halves of rotational prolate ellipsoids^6^. The respective dimensions were estimated from calibrated photographs by image analysis (cellSens Dimension 1.7.1; Olympus Soft Imaging Solutions, Münster, Germany). The relationship between mass and the measured surface area was plotted and an empirical regression model was fitted. Data from a compilation between different cultivars and development stages ranging from green fruitlets to fully mature fruit were used (see supplementary information). The total number of individual fruit replications was 200.

#### Identifying high flux pathways for water movement

Preferential pathways of water movement through the surface of ‘Sonsation’ fruits were identified by selectively sealing different portions of the fruit surface with silicone rubber. Two experiments were carried out with the rates of osmotic water uptake and transpirational water loss being quantified gravimetrically.

The first experiment was conducted in two phases (‘control’/’treatment’) using a repeated-measures design, comparing each treatment with its control (Fig. 4, Table 1). In phase I, fruit with calyx and peduncle (cut to 5 mm long) was incubated for 1.5 h. Rates of water movement were determined gravimetrically as described above. There was no sealing in phase I (‘control’). In phase II, selective sealing using silicone rubber or cutting was applied as follows (‘treatment’): (1) sepals (including episepals) and stamina were excised at their base and the cut surfaces, the calyx-receptacle junction, and the peduncle end were sealed (Fig. 3); (2) sepals and stamina were excised and the cut surfaces, the calyx-receptacle junction, and the peduncle end were left un-sealed, and (3) the peduncle end was sealed (Table 1). Each treatment had its own control. Fruit were held in a controlled temperature room for 1.5 h for the silicone rubber to cure. Fruits were then incubated for an additional 1.5 h.

In the second experiment, the portion of the fruit surface responsible for the increased water movement in the calyx region was identified by sealing a progressively increasing portion of the fruit surface—beginning at the proximal end of the fruit (Fig. 3, Table 2). The treatments were (a) ‘Control’: no sealing; (b) ‘Sepals sealed’: the sepals and the peduncle surface were sealed. The petal and stamina abscission zones, the calyx-receptacle junction, and the seedless zone of the receptacle were not sealed; (c) ‘Sepals, petal and stamina abscission zones and calyx-receptacle junction sealed’: the sepals, abscission zone, stamen, peduncle, and calyx-receptacle junction was cut and all surfaces sealed (the ‘seedless’ zone, strictly, the zone lacking the ‘pips’ or single-seeded achenes, was not sealed); and (d) ‘Seedless zone sealed’: the seedless zone of the receptacle was sealed, also the abscission zone and stamens, the calyx-receptacle junction, the peduncle, and sepals were removed and sealed. The cut end of the peduncle was sealed in all these treatments including the control.

Rates of transpiration and osmotic water uptake were calculated as described above. The total number of replications per treatment was 30. From these treatments, the amounts of water transpired or taken up through the sepals and through the peduncle surface or through the abscission zones of petals, stamina, the junction between calyx and receptacle, and through the seedless zone and through the receptacle bearing seeds were obtained by calculating the difference between the respective rates of water movement in phase I and phase II (Table 3).

To identify the sites of water uptake microscopically, ‘Sonsation’ fruits from the same batch were incubated in 0.1% (w/w) aqueous acridine orange (Carl Roth, Karlsruhe, Germany) for 1.5 h. The cuticle is impermeable to the fluorescent tracer acridine orange. The dye therefore penetrates only in regions where the cuticle barrier is bypassed. Fruits were rinsed with deionized water, blotted using soft tissue paper, and viewed under a fluorescence binocular microscope (MZ10F; Leica Microsystems, Wetzlar, Germany) (Figs. 5, 6).

To establish whether the same preferential pathways for water movement occurred in other strawberry cultivars, the relative contributions to water movement of the various pathways—through the petal and staminal abscission zones, and around the junction between calyx and receptacle, were determined on a whole fruit basis in ‘Clery’, ‘Sonsation’, ‘Florentina’, ‘Dream’, ‘Joly’, and ‘Elsanta’ (Table 4). To do this the abscission zone and the junction were sealed using silicone rubber. Unsealed fruit served as controls. Pathways of water movement were also viewed by fluorescence microscopy. Fruits from the same batch were incubated in a solution of the fluorescence tracer acridine orange at 0.1% (w/w) (Carl Roth, Karlsruhe, Germany) for 1.5 h. Thereafter, fruits were observed under a fluorescence binocular microscope (MZ10F; Leica Microsystems, Wetzlar, Germany).

#### External factors affecting water movement

##### Effect of temperature

The effects of temperature (T) on osmotic water uptake and transpiration were investigated in ‘Laetitia’ strawberry (Fig. 7b,c). Fruits were incubated at 2, 12, 23, or 35 °C. The rates of water movement were calculated as described above. Thereafter, the rates of transpiration were normalized by dividing by the gradient in water vapor concentration between the inside of the fruit (assumed to be saturated) and the outside atmosphere at the incubation temperature. Because in the transpiration assays the fruit was incubated above dry silica gel and the water vapor concentration above the dry silica gel is practically zero, the driving force for transpiration equals the water vapor concentration at saturation at the respective temperature. The values so obtained will be proportional to the fruit’s permeance.

##### Effect of relative humidity

The effect of relative humidity (RH) on the rate of transpiration in ‘Laetitia’ strawberry was determined by incubating the fruit at ~ 0, 30, 75, or 92% RH and 22 °C using silica gel, or saturated solutions of CaCl_2_, NaCl, or KNO_3_, respectively (Fig. 7a)^25^. Rates of transpiration were normalized by dividing by the gradient in water vapor concentration between the atmosphere inside the fruit (assumed to be saturated) and the outside atmosphere.

##### Effect of storage duration

The effect of storage duration of ‘Clery’ fruit on water movement was investigated in two experiments. In the first, fruits were held in polyethylene (PE) boxes above saturated NaCl (Carl Roth, Karlsruhe, Germany) (RH ~ 76%^25^) at 2 °C for 0, 1, 2, 4, 7, or 10 d (Fig. 8). Fruits were placed in the boxes on foam trays over a metallic mesh to avoid direct contact with the NaCl. The mass loss during storage was determined by weighing fruit before and after the storage period. Thereafter, fruits were equilibrated at room temperature and ~ 40% RH for 2 h before the rates of transpiration and osmotic water uptake were measured as described above.

Second, the effect of inducing a mass loss by incubating ‘Clery’ fruit for 24 h at different RHs on rates of osmotic water uptake and transpiration was determined (Fig. 9). To induce the mass loss, fruits were pre-incubated in PE boxes above silica gel (0% RH), or above saturated solutions of CaCl_2_ (30% RH), NaCl (75% RH), or deionized water (100% RH)^25^. The mass loss was determined by weighing fruit before and after the pre-incubation period. The rates of transpiration and water uptake after mass loss induction were determined gravimetrically.

### Data analyses

All experiments comply with relevant institutional, national, and international guidelines and legislation. Data were analyzed by analysis of variance (ANOVA) and linear regression. Means were compared using Dunnett’s and Tukey’s tests (p < 0.05) in the statistical software R (version 4.1.0; R Foundation for Statistical Computing, Vienna, Austria). Data are presented as means ± standard errors.

## Supplementary Information

Supplementary Information.

## Data Availability

The datasets generated during the current study are available from the corresponding author upon reasonable request.
